# Bone mineral density in patients with juvenile systemic lupus erythematosus

**DOI:** 10.1186/1546-0096-10-S1-A27

**Published:** 2012-07-13

**Authors:** Ana Paula Roenick Guenka, Blanca Elena Rios Gomes Bica, Laura Maria Carvalho de Mendonça, Maria Lucia Fleiuss de Farias, Lucas Pinheiro Aversa de Escobar Velloso, Mario Newton Leitão de Azevedo

**Affiliations:** 1 Universidade Federal do Rio de Janeiro, Rio de Janeiro, Brazil

## Purpose

Evaluate the bone mass in a group of patients diagnosed with juvenile systemic lupus erythematosus (JSLE), which carry high risks of developing low bone mass, as well as verifying alterations on calcium metabolism.

## Methods

Cross-sectional study, in which were evaluated 53 patients from the Rheumatology ambulatory in HUCFF/ UFRJ, diagnosed with JSLE in their childhood or teenage years, according to ACR (American College of Rheumatology) criteria. Subjects were submitted to a single blood and urine collection for dosing of the following variables: alkaline phosphatase, serum phosphorus and calcium, urea, creatinine, erythrocyte sedimentation rate (ESR), intact PTH, free T4, TSH, 24-hour calciuria. Bone density was measured from lumbar spine, and full body and/or total femur, according to age range, using DEXA methods and Prodigy Advance equipment. Also, medical records were analyzed collecting the following data: age, sex, time coping with the disease, body mass index (BMI), disease progression with SLEDAI average for the last 6 months, number of diagnostic criteria, cumulative dose of 3 drug-induced osteoporosis classes (glucocorticoid, methotrexate and anticoagulant). Finally, the collected data was submitted to statistic analysis. Patients suffering from renal insufficiency or currently on bone antiresorptive or bone formation stimulators drugs were excluded.

## Results

92% of patients were females, being only 4 male subjects. Analyzed parameters averages, and their corresponding standard deviation (SD): Age – 21 years old (4,0); time coping with the disease – 7,9 years (5,7); number of ACR criteria at diagnosing moment – 5,6 (1,4); BMI – 23,2 kg/m^2^ (4,7); SLEDAI – 3,7 (4,6) R=1,23. Not only was the accumulative glucocorticoid dosage calculated, but also the prednisone equivalency. The maximum used dosage was 133,88g, with an average of 29,1g and standard deviation of 27,8 (R=1,0). The only anticoagulant used was warfarin, with maximum dosage of 39,76g, average of 1,3g and standard deviation of 5,9 (R=4,5). Maximum dosage for methotrexate was 2,49g, average of 0,2g and standard deviation of 0,6 (R=2,6). 15 patients presented densitometry alterations, characterized by Z-score lower or equal to 2 standard deviation. No clinically significant laboratorial alterations on calcium metabolism were found.

## Conclusion

No direct or inverse linear relation (correlation) was observed between the analyzed parameters and densitometry findings according to the Z-score.

## Disclosure

Ana Paula Roenick Guenka: None; Blanca Elena Rios Gomes Bica: None; Laura Maria Carvalho de Mendonça: None; Maria Lucia Fleiuss de Farias: None; Lucas Pinheiro Aversa de Escobar Velloso: None; Mario Newton Leitão de Azevedo: None.

